# Enhanced Survival of High-Risk Medulloblastoma-Bearing Mice after Multimodal Treatment with Radiotherapy, Decitabine, and Abacavir

**DOI:** 10.3390/ijms23073815

**Published:** 2022-03-30

**Authors:** Marieke Gringmuth, Jenny Walther, Sebastian Greiser, Magali Toussaint, Benjamin Schwalm, Marcel Kool, Rolf-Dieter Kortmann, Annegret Glasow, Ina Patties

**Affiliations:** 1 Department of Radiation Oncology, University of Leipzig, Stephanstraße 9a, 04103 Leipzig, Germany; marieke.gringmuth@koeln.de (M.G.); rolf-dieter.kortmann@medizin.uni-leipzig.de (R.-D.K.); annegret.glasow@medizin.uni-leipzig.de (A.G.); 2 Fraunhofer Center for Microelectronic and Optical Systems for Biomedicine, Herman-Hollerith-Straße 3, 99099 Erfurt, Germany; jenny-walther1@web.de (J.W.); sebastian.greiser@izi.fraunhofer.de (S.G.); 3 Fraunhofer Institute for Cell Therapy and Immunology, Perlickstraße 1, 04103 Leipzig, Germany; 4 Department of Neuroradiopharmaceuticals, Institute of Radiopharmaceutical Cancer Research Helmholtz-Zentrum Dresden-Rossendorf (HZDR), Research Site Leipzig, Permoserstraße 15, 04318 Leipzig, Germany; m.toussaint@hzdr.de; 5 Hopp Children’s Cancer Center (KiTZ), Im Neuenheimer Feld 430, 69120 Heidelberg, Germany; b.schwalm@kitz-heidelberg.de (B.S.); m.kool@kitz-heidelberg.de (M.K.); 6 Division of Pediatric Neurooncology, German Cancer Research Center (DKFZ) and German Cancer Research Consortium (DKTK), Im Neuenheimer Feld 280, 69120 Heidelberg, Germany; 7 Princess Máxima Center for Pediatric Oncology, Heidelberglaan 25, 3584 CS Utrecht, The Netherlands

**Keywords:** medulloblastoma, radiation, decitabine, abacavir, in vivo study, magnetic resonance imaging, T2 mapping, bioluminescence imaging, gene expression microarray

## Abstract

Children with high-risk SHH/*TP53*-mut and Group 3 medulloblastoma (MB) have a 5-year overall survival of only 40%. Innovative approaches to enhance survival while preventing adverse effects are urgently needed. We investigated an innovative therapy approach combining irradiation (RT), decitabine (DEC), and abacavir (ABC) in a patient-derived orthotopic SHH/*TP53*-mut and Group 3 MB mouse model. MB-bearing mice were treated with DEC, ABC and RT. Mouse survival, tumor growth (BLI, MRT) tumor histology (H/E), proliferation (Ki-67), and endothelial (CD31) staining were analyzed. Gene expression was examined by microarray and RT-PCR (Ki-67, VEGF, CD31, CD15, CD133, nestin, CD68, IBA). The RT/DEC/ABC therapy inhibited tumor growth and enhanced mouse survival. Ki-67 decreased in SHH/*TP53*-mut MBs after RT, DEC, RT/ABC, and RT/DEC/ABC therapy. CD31 was higher in SHH/*TP53*-mut compared to Group 3 MBs and decreased after RT/DEC/ABC. Microarray analyses showed a therapy-induced downregulation of cell cycle genes. By RT-PCR, no therapy-induced effect on stem cell fraction or immune cell invasion/activation could be shown. We showed for the first time that RT/DEC/ABC therapy improves survival of orthotopic SHH/*TP53*-mut and Group 3 MB-bearing mice without inducing adverse effects suggesting the potential for an adjuvant application of this multimodal therapy approach in the human clinic.

## 1. Introduction

Medulloblastoma (MB) is one of the most common malignant brain tumors in children with incidence peaks at 1–9 years [1]. In 2021, the World Health Organization defined four molecular subgroups (WNT-activated; SHH-activated and *TP53*-wildtype; SHH-activated and *TP53*-mutant; non-WNT/non-SHH, comprising Group 3 and 4), whereby studies showed a complex clustering of subtypes within the MB subgroups especially of i.e., Group 3 and 4 [2,3,4].

Overall survival (OS) differs greatly between MB types. Thereby, children > 4 years with sonic hedgehog- (SHH-) activated and *TP53*-mutated MB (5-y OS 43%) and patients with *MYC*-amplificated non-WNT/non-SHH MB (5-y OS 42%) have the worst prognosis [2,3,5].

Therefore, the improvement of survival has priority when initiating new trials in these patient cohorts. The European Society for Pediatric Oncology (SIOP) currently treats such high-risk (HRMB) patients according to the SIOP-HRMB protocol including surgery, followed by induction chemotherapy (CT) (etoposide, carboplatin) and conventional/hyperfractionated radiation therapy (RT) or high-dose CT (thiotepa) and conventional RT, combined with maintenance CT (standard: vincristine (VCR)/lomustine (CCNU)/cisplatin alternating with VCR/cyclophosphamide, or temozolomide) for children > 3 years [6]. A former phase I/II trial of the US Children’s Oncology Group investigated a therapy consisting of conventional RT + CT (carboplatin/VCR) and maintenance CT (cyclophosphamide/VCR) with or without cisplatin and found no cisplatin-induced survival benefit [7]. A recently published US phase III trial where high-risk MB patients received conventional RT combined with CT (VCR) with or without carboplatin followed by maintenance CT (cisplatin/cyclophosphamide/VCR) with or without isotretinoin revealed a 19% improved event-free 5-year survival in carboplatin-treated high-risk Group 3 MB patients although an increased risk of acute adverse effects was observed [8]. 

The intensive treatment is associated with a higher risk for long-term neurocognitive, behavioural, and emotional deficits (reviewed by [9,10]), especially in children treated at younger age [11]. Such long-term adverse effects include decreased intelligence, difficulties with information processing, vocal skills, and memory loss as well as progressive loss of focus [12,13,14]. Whole brain irradiation is a high-risk factor due to the radiation-induced reduction of hippocampal neurogenesis [15,16] and neuroinflammation [15,17], both asssumed to be responsible for neurocognitive disorders. In infants, previous trials attempted to substitute whole brain irradiation with focal radiation, intracranial methotrexate treatment (MTX) or high-dose chemotherapy (HDCT), but have not yielded enhanced overall survival although being better tolerated and avoiding serious radiation-induced neurologic adverse events [11,18,19,20]. Nevertheless, chemotherapy, especially intracranial application of MTX or HDCT, is also reported to induce massive cognitive effects [9,21,22,23,24].

Novel therapeutic approaches are urgently needed to enhance the overall survival especially in the high-risk MB patients, without compounding chemo- and radiotherapy-induced adverse effects.

Aberrant gene promotor methylation frequently occurs in MBs and may silence tumor suppressor genes (TSG) [5,25,26,27,28] which can be reactivated by demethylating agents such as 5-aza-2′deoxycytidine (decitabine, DEC) [26,29,30,31]. In addition, DEC causes cell death directly by induction of DNA double-strand breaks [31,32,33]. Since DEC is a nucleoside analogue incorporated into the DNA during S phase of dividing cells, fast proliferating tumor cells would be killed preferably [34]. 

The majority of MBs has an enhanced telomerase activity [35] or human telomerase reverse transcriptase (*hTERT)* mRNA expression [36]. This can be caused by *hTERT* promotor mutations or by upstream of the transcription start site (UTSS) hypermethylation [37,38,39]. Thereby, telomerase-activating mutations occur most frequently in SHH-activated MBs [5,40] showing an age-dependent incidence of 34% in children SHH and of 73% in adult SHH [39]. In contrast, *hTERT* promotor hypermethylation was demonstrated in all MB subgroups with an incidence of 69% in Group 3 and 36% in SHH MB. This suggests that inhibition of telomerase activity would be a promising approach to block telomerase-dependent aberrant tumor cell proliferation in these MB subtypes. Abacavir (ABC) is a telomerase inhibitor that can trespass the blood-brain-barrier [41] and suppresses telomerase activity both, by inhibiting reverse transcriptase and by down-regulating *hTERT* promoter [42]. Additionally, ABC leads to proliferation inhibition and MB cell differentiation and causes direct MB cell cytotoxicity by induction of DNA double-strand breaks [33,42].

Previously, we have shown that multimodal treatment combining the de novo methyltransferase inhibitor DEC, the telomerase inhibitor ABC, and radiation therapy (RT) could significantly reduce clonogenic survival in three different medulloblastoma cell lines (DAOY, MEB-Med8a, D283-Med) [33]. Importantly, this multimodal therapy did not impair the number of neural progenitor cells in murine hippocampal slice cultures. Thus, the combination of DEC, ABC, and RT seemed to be promising to treat medulloblastoma while avoiding serious neurologic adverse effects. In this preclinical study, we investigated if this innovative strategy is also effective in vivo using orthotopic patient-derived xenograft (PDX) models for a SHH/*TP53*-mutated (SHH/*TP53*-mut) and a non-WNT/non-SHH, *MYC*-amplificated Group 3 (Group 3) MB tumor, representing two high-risk MB subtypes.

The primary endpoints were (I) mouse survival, which was defined by the time from tumor cell implantation to euthanasia and (II) tumor growth, which was measured by optical (bioluminescence, BLI) and magnetic resonance imaging (MRI). Both imaging techniques were correlated. Tumor tissue was analyzed by immunostaining and real-time PCR for altered proliferation index, vascularity, stemness, and immune cell invasion/activation after therapy. Gene expression microarray analyses were performed to examine changes in the expression of gene sets and single genes after therapy.

## 2. Results

### 2.1. Optimization of MRI Volumetry Measurements

**Figure 1:**
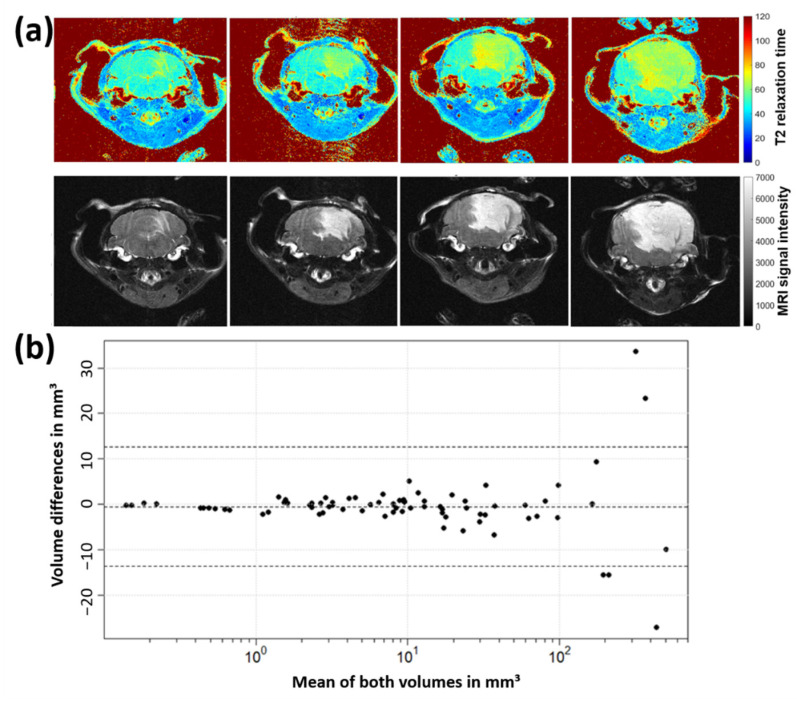
(**a**) Medulloblastoma growth progress (animal 1972, weeks 3, 5, 6 and 7 post tumor cell inoculation) in both contrast methods—T2 average image and T2 map. (**b**) Bland-Altman plot to compare differences of tumor volumes determined from T2 average images and T2 maps. The tumor volume identified in T2 average images was subtracted from the corresponding T2 map volume for each measurement.

Comparative measurements using T2 map and T2 average contrast method were conducted to ascertain the most sensitive method for volumetric calculations. Figure 1a shows representative MRI slices of one tumor-bearing mouse at different time points using T2 map and T2 average contrast method. For T2 average images (as well as any directly acquired multi-slice multi-echo images (PD- and T2-weigthed)), MRI signals from tumor regions were hyperintense compared to healthy brain tissue. T2 maps showed T2 relaxation times around 47 ms for healthy brain tissue, 68 ms for medulloblastoma structures and 125 ms for cerebrospinal fluid (ventricles). Tumor tissue showed smooth boundaries to the surrounding brain and appeared more perforated in T2 maps than in T2 average images.

However, the T2 map contrast method emerges less sensitive for the here examined medulloblastoma subtype. A cohort of eight mice was imaged in 76 MRI sessions where T2 average contrast reveals 430 tumor-containing slices and T2 map contrast method 394. Calculated tumor volumes measured in T2 average images were on average marginally higher than those determined in corresponding T2 maps. To compare the tumor volumetry data directly, corresponding volumes of T2 average images and T2 maps were plotted using the Bland-Altman method (Figure 1b). Volume differences uniformly scattered around zero and rarely exceeded the lower or upper reference line (mean ± 1.96 ∙ standard deviation), hence no significant difference of medulloblastoma volumes determined from T2 average images and T2 maps can be assumed. Differences increased with rising mean tumor volumes, which results from higher segmentation uncertainties for more complex and larger image structures. T-test yielded that mean volume differences of both contrast methods being zero cannot be rejected (t(75) = −0.75, *p* = 0.46), which confirms the assumption according to the Bland-Altman plot.

### 2.2. Dose-Finding and Toxicity Studies

#### 2.2.1. Whole Brain Irradiation (WBI)

For each MB model a preliminary dose-finding study was implemented where mice were single-dose irradiated at day 28 post tumor cell implantation. The radiation tolerance (adverse effects) and the time until the mice had to be euthanized (survival time) were analyzed.

In the Group 3 MB model, WBI doses of 2, 5, and 10 Gy were tested using 6 mice per radiation dose. Mice, which received 5 and 10 Gy revealed a diminished general condition accompanied by weight loss 5–7 days after WBI. WBI with 2 Gy induced no adverse effects and enhanced the median survival time by 16% (not significant, data not shown). The combination studies in the Group 3 MB model were therefore conducted using the 2 Gy dose. Results from main experiments of Group 3 led to further optimization tests in the second model. To enhance the WBI-induced survival benefit towards significant values devoid of adverse effects, WBI doses of 3 and 4 Gy were applied to SHH/*TP53*-mut mice (3 mice per radiation dose) in a preliminary experiment. Both doses (3, 4 Gy) induced no adverse effects and 4 Gy enhanced the median survival time significantly by 25% (log rank test *p* = 0.025; data not shown). The combination studies in the SHH/*TP53*-mut MB model were therefore conducted using the 4 Gy dose.

#### 2.2.2. Decitabine and Abacavir

To evaluate the systemic toxicity of decitabin (DEC) and abacavir (ABC), 3 concentrations of each drug with 3 mice per concentration were used. Only Group 3 MB-bearing mice were implemented in this study. As the tolerance to DEC and ABC is dependent on the mouse strain, but not on the tumor subtype, no further studies were conducted using the SHH/*TP53*-mut MB model. The intraperitoneal application started at day 21 after tumor cell implantation. Mice got DEC (0.1, 0.5, 1 mg/kg) or ABC (10, 20, 50 mg/kg) daily for 14 days. Livers of 38 mice with various treatments were inspected for organ toxicity. Thereby, no abnormalities could be found.

DEC at higher concentrations (0.5 and 1 mg/kg/d) revealed a diminished general condition accompanied by weight loss and led to the premature euthanasia of these mice. The administration of 0.1 mg/kg/d DEC was well tolerated and was able to prolong the survival time until euthanasia by 30% (not significant, data not shown). The combination studies were therefore conducted using 0.1 mg/kg/d DEC. 

ABC revealed no adverse effects on the general health condition of the mice. The highest concentration of 50 mg/kg/d even enhanced the median survival time until euthanasia by 30% (not significant, data not shown). The combination studies were therefore conducted using 50 mg/kg/d ABC.

### 2.3. Enhanced Survival of Mice after Multimodal Therapy

For Group 3 MB studies, in total 83 mice were stereotactically transplanted. Three mice died during surgery. 1/80 had to be euthanized during treatment due to poor health, 79 mice received the treatment as planned. 71/79 had to be euthanized because of tumor-driven human endpoints, 2/79 died overnight, 6/79 survived without clinical symptoms and were euthanized 139 days after surgery (3-time median survival time). Macroscopic tumor bearing was infrequent and seen in 35/79 animals. By histological analyses 77/79 mice (incl. 4/6 long-time survivors) were found tumor-bearing and were included in survival analysis (Table 1). 

**Figure 2:**
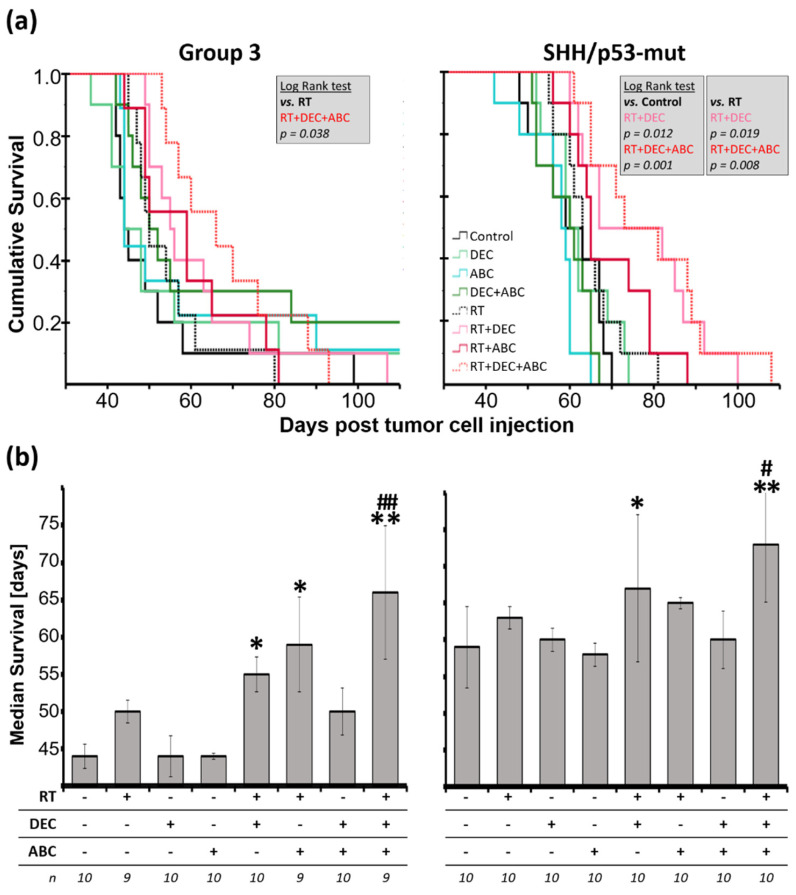
(**a**) Cumulative survival and (**b**) Median survival of Group 3 and SHH/*TP53*-mut medulloblastoma-bearing mice treated with radiation therapy (RT; Group 3: 2 Gy, SHH/*TP53*-mut: 4 Gy), decitabin (DEC; 0.1 mg/kg/d) and/or abacavir (ABC; 50 mg/kg/d). (**a**) Kaplan-Meier analyses and significant log-rank test results. (**b**) Data presented are medians of survival ± standard error. Statistical significance calculated by two-sided Mann-Whitney is indicated by asterisks (*, *p* ≤ 0.05; **, *p* ≤ 0.01; vs. sham-treated control) or by hashtag (#, *p* ≤ 0.05; ##, *p* ≤ 0.01; vs. radiation-treated group). Numbers of animals included are given below (n).

For SHH/*TP53*-mutated MB studies, in total 90 mice were stereotactically transplanted. One mouse died during surgery. All mice were checked for successful tumor cell implantation by BLI one week after surgery. One experiment (8 animals) and also one mouse of another experiment had to be excluded completely as no positive BLI signal was present, and therefore successful tumor implantation could not be validated. All remaining 80 animals were tumor-bearing and included in survival analyses (Table 1). In Kaplan-Meier analyses, multimodal treatment with RT/DEC/ABC and combined RT/DEC treatment significantly prolonged survival compared to sham-treated SHH/*TP53*-mut MB-bearing animals (Figure 2a).

Also, compared to the irradiated control group, the combined RT/DEC/ABC (both subtypes) and RT/DEC (SHH/*TP53*-mut MB only) treatment showed a significant survival benefit. The median survival after multimodal treatment thereby was enhanced by 50% in Group 3 MB (44 ± 1.6 d vs. 66 ± 8.9 d) and 24% in SHH/*TP53*-mut MB (59 ± 5.5 d vs. 73 ± 7.9 d) compared to sham-treated control animals and 16% (SHH/*TP53*-mut) and 32% (Group 3) compared to RT-treated mice (Figure 2b). Similar to Kaplan-Meier analyses, the RT/DEC therapy showed a significant increase in median survival by 25% (Group 3) and 14% (SHH/*TP53*-mut). In Group 3 MB-bearing animals, also the combined RT/ABC therapy significantly enhanced the median survival by 34%. RT alone has no significant effect in both MB models, however, 2 Gy WBI in Group 3 MB-bearing animals (50 ± 1.5 d; 14% enhancement) seemed to be more effective than 4 Gy WBI in SHH/*TP53*-mut MB-bearing animals (63 ± 1.5 d; 7% enhancement).

### 2.4. Inhibition of Tumor Growth after Multimodal Therapy in SHH/TP53-Mut MB

**Figure 3:**
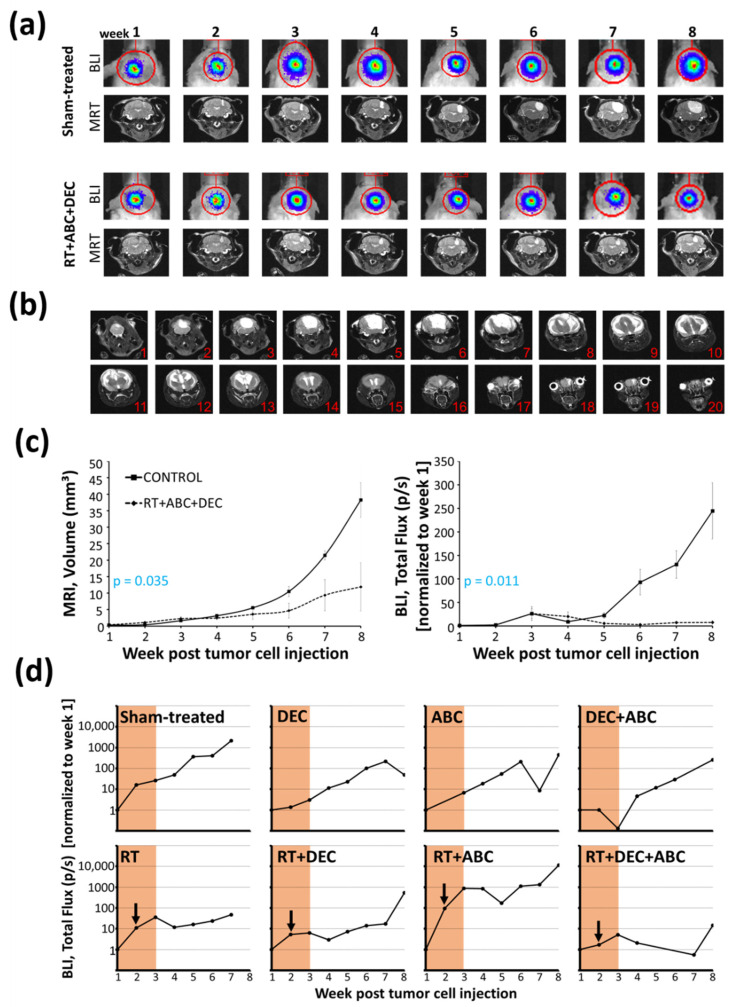
Tumor growth measured by magnetic resonance (MRI) and bioluminescence imaging (BLI) of SHH/*TP53*-mut MB-bearing mice treated with radiation therapy (RT), decitabin (DEC) and abacavir (ABC). (**a**) Representative T2-weighted average MRI (slice 5 or 6) and corresponding BLI images of sham- and multimodal-treated (RT/DEC/ABC) mice. (**b**) T2-weighted average MRI images (slice number from occipital to rostral) of one representative sham-treated mouse 64 d post tumor cell injection (3 d before euthanasia). (**c**) Tumor volume determined by MRI (left) and total flux of bioluminescent tumor cells determined by BLI (right) of sham- and multimodal-treated mice. Data presented are means ± SEM, n = 4. *p*-values were calculated by one-way ANOVA with tumor volume/total flux as dependent and treatment (yes/no) as independent variable. (**d**) Representative progress of total flux over eight weeks post tumor cell injection measured by BLI of one study group including one mouse each treatment. Treatment period of 14 d and radiation time point at day 8 is marked.

Tumor growth of SHH/*TP53*-mut MB was measured non-invasively in four mice of each, sham- and multimodal-treated group, by MRI and BLI in parallel (Figure 3a,c). A significant retardation in the RT/DEC/ABC compared to the control group was observed. Only measurements of week 1–8 post tumor cell implantation not including liquid- or tumor mass-infiltrated brain ventricles were analyzed. The maximum tumor size and relative total flux at week 8 were 38 ± 5 mm^3^ and 245 ± 60 (absolute total flux: 2.2 × 10^7^ ± 0.8 × 10^7^ p/s).

By MRI, further tumor enlargement and the infiltration of the central and lateral ventricles could be observed starting at week 9 in the sham-treated animals (one week before euthanasia at the latest), (Figure 3b). Ventricle compression and liquor stasis, resulted in substantial distension in all spatial directions. Central aqueduct and fourth ventricle were displaced into the left hemisphere causing asymmetry in the lateral ventricles as well. This presented in the MRI by both a larger ventricle structure in single sections as well as ventricles appearing in higher number of sections each week. The continuous inflation led to bulging of the skull bone structure and persistent separation of the sagittal suture, causing the symptom of a “bulky”-rounded head and providing a temporary pressure relief. 

In Figure 3d, the total flux values of one experimental study group (8 mice; 1 mouse per treatment group) are depicted over 8 weeks post tumor cell transplantation. The treated, and in particular the RT-treated mice (except of RT+ABC), showed a flatter increase of total flux compared to the sham-treated mouse. The multimodal therapy with RT/DEC/ABC induced even a plateau in tumor growth until week 7.

### 2.5. Strong Correlation of BLI and MRI Data

**Figure 4:**
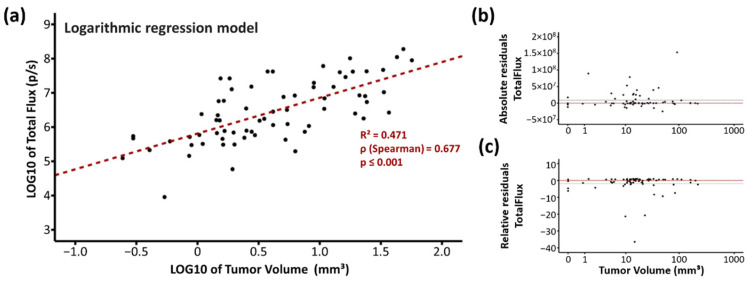
Correlation analyses of magnetic resonance (MRI) and bioluminescence imaging (BLI) using the logarithmic regression model. (**a**) LOG/LOG diagram of MRI tumor volume versus BLI total flux. Each point indicates one measurement, n = 74. Coefficient of determination (R^2^), Spearman’s rank correlation coefficient (ρ) and *p* value determined by ANOVA are given in the chart. (**b**) Absolute and (**c**) relative residuals of total flux. The dotted red line and the solid black line indicate the zero value and the mean value of residuals.

To correlate the MRI-based tumor volume and the BLI-based total flux values, the logarithmic regression model was used (Figure 4a–c). By plotting the log10 values of both parameters, a linear regression curve y = 5.81 + 1.04·x fits the values best with a coefficient of determination R^2^ = 0.471, which means that 47.1% of the total flux variance can be explained by the tumor volume variance. The Spearman’s rank correlation coefficient of ρ = 0.677 indicates a strong positive correlation (*p* ≤ 0.001).

The absolute residuals of total flux were shifted slightly greater than zero suggesting that total flux likely overestimated the tumor volumes (Figure 4b). The relative residuals of total flux were similar distributed over the whole tumor volume range indicating that there is no association between overestimation of total flux and a smaller/greater tumor volume (Figure 4c).

### 2.6. Tumor Histology

**Figure 5:**
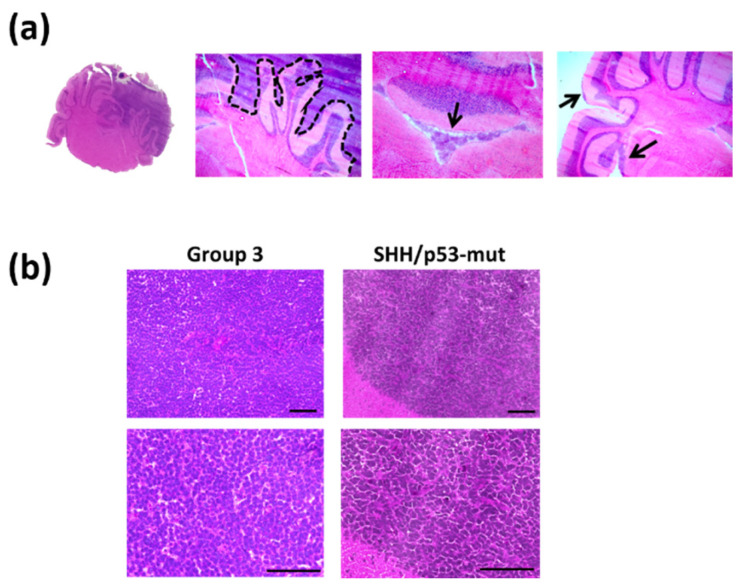
Histology of Group 3 and SHH/TP53-mut orthotopic medulloblastoma. Brain slices were stained by hematoxylin/eosin. (**a**) Histological characteristics of orthotopic Group 3 and SHH/*TP53*-mut medulloblastoma, scale bars = 50 µm. (**b**) Expansion of tumor growth on the mouse brain. Brain slice with tumor tissue within the right cerebellum; dashed line marks the tumor border; arrows mark tumor cells within the 4th ventricle and on the brain exterior (from left to right image).

To evaluate the tumor spread within the brain and the histological tumor subtype, hematoxylin/eosine stainings were performed. The orthotopic tumors grew brain displacing and invasive, tumor cells were found within the 4th ventricle and on the brain exterior (Figure 5a).

Both MB subtype models showed no therapy-induced change in morphology (Figure 5b).

### 2.7. Reduced Tumor Proliferation Index after Multimodal Therapy in SHH/TP53-Mut MB

**Figure 6:**
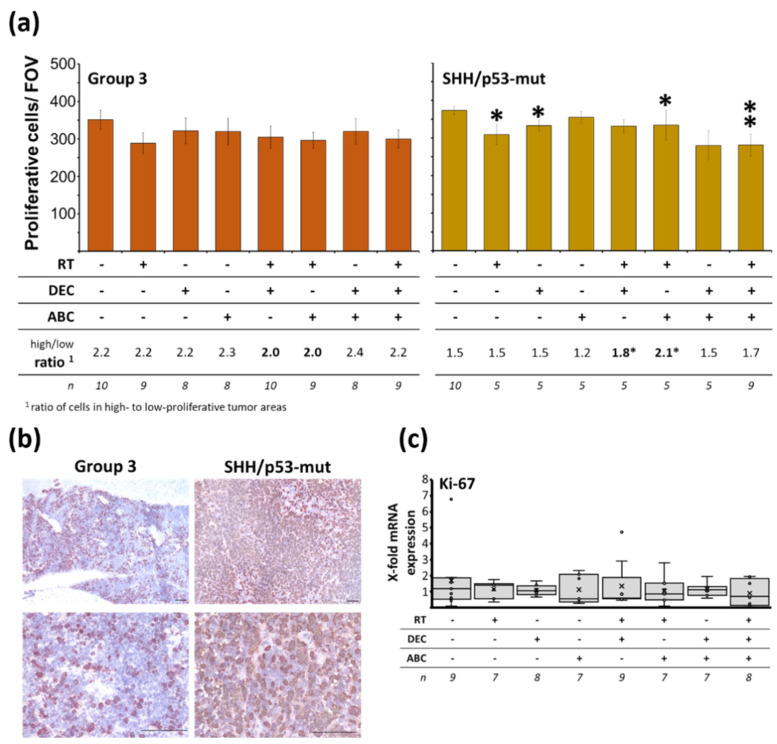
Proliferating cells within Group 3 and SHH/TP53-mut othotopic medulloblastomas dependent on treatment of the MB-bearing mice with radiation therapy (RT), decitabine (DEC), and/or abacavir (ABC). (**a**) Proliferative cells/field of view (FOV), mean of high- and low-proliferative tumor areas. Data presented mean ± SEM, numbers of analyzed animals/treatment group and ratio of cell numbers in high- to low-proliferative areas is given below. Statistical significance was determined by two-sided Mann-Whitney test (*, *p* ≤ 0.05; **, *p* ≤ 0.01; versus sham-treated control). (**b**) Representative photographs of Ki-67/AEC-stained proliferative cells in Group 3 and SHH/*TP53*-mut tumors, lower panel shows low-proliferative areas at 400-fold magnification, scale bars = 100 µm. (**c**) Ki-67 mRNA expression in Group 3 MB tissue determined by RT-PCR. Data are presented as box-and-whisker plots, numbers of analyzed animals/treatment group are given below (n).

To investigate the effect of therapy on the proportion of proliferating tumor cells, Ki-67 AEC staining and qRT-PCR was conducted. In the sham-treated control group, Group3 and SHH/*TP53*-mut tumors showed a similar quantity of proliferative cells (32.6 and 36% of all cells; cells per FOV: 351.33 ± 25.6 and 373.23 ± 10.9; n = 10), (Figure 6a,b). Distribution of proliferative cells was inhomogeneous, showing high- and low-proliferative areas in Group 3 tumors. The proliferative cell count ratio between high- and low-proliferative areas was significantly higher in Group 3 MBs compared to SHH/*TP53*-mut tumors (2.2 ± 0.2 vs. 1.5 ± 0.1, n = 10, *p* ≤ 0.01).

A significant reduction of proliferative cells per FOV to 84.2 ± 4, 91.6 ± 4, 78.5 ± 13, and 75.6 ± 8% (n = 9; *p* ≤ 0.05) could be observed in SHH/*TP53*-mut MB only after RT alone, DEC alone, combined RT/ABC, and multimodal treatment with RT/DEC/ABC (Figure 6a). Also, in Group 3 tumors a trend to decreased proliferation was seen in these groups without reaching significance. The proliferative cell count ratio significantly changed to more high-proliferative areas only in SHH/*TP53*-mut MBs after RT/DEC and RT/ABC treatment (control: 1.5 vs. RT/DEC: 1.8, RT/ABC: 2.1). Ki-67 mRNA expression levels in Group 3 MB remained unchanged after therapy (Figure 6c).

### 2.8. Reduced Tumor Vascularization after Multimodal Therapy in SHH/TP53-Mut MB

**Figure 7:**
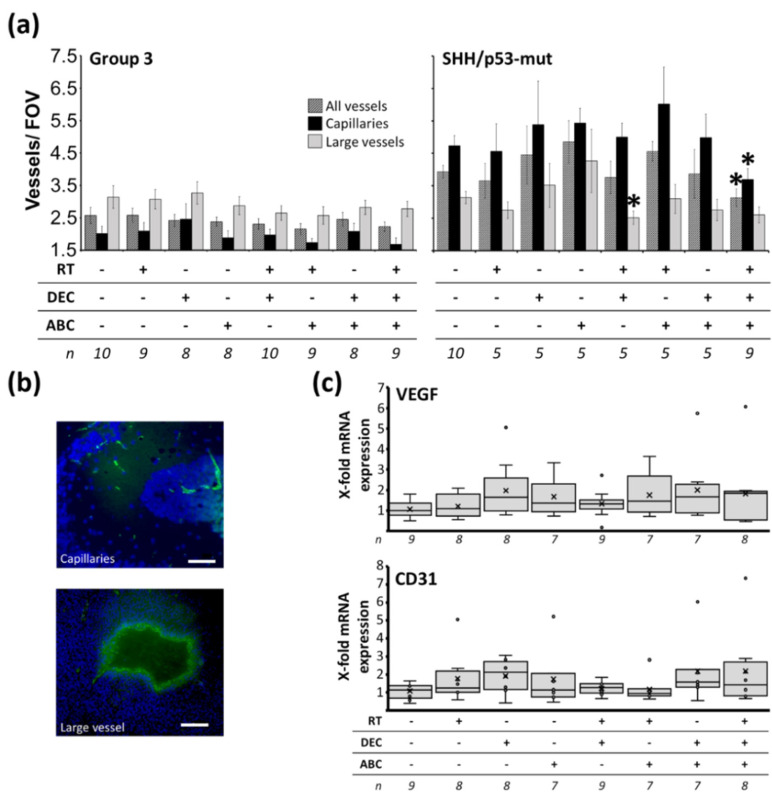
Vascularization in Group 3 and SHH/*TP53*-mut orthotopic medulloblastomas dependent on treatment of the MB-bearing mice with radiation therapy (RT), decitabine (DEC), and/or abacavir (ABC). (**a**) Capillaries and larger vessels per field of view (FOV). Data presented mean ± SEM, numbers of analyzed animals/treatment group is given below. Statistical significance was determined by two-sided Mann-Whitney test (*, *p* ≤ 0.05 versus sham-treated control). (**b**) Representative photographs of CD31-stained vascular cells (capillaries and large vessel) in Group 3 and SHH/*TP53*-mut tumors, scale bars = 100 µm. (**c**) CD31 and VEGF mRNA expression in Group 3 medulloblastoma tissue determined by RT-PCR. Data are presented as box-and-whisker plots, numbers of analyzed animals/treatment group are given below (n).

To assess tumor vascularization and/or therapy-induced changes in vascular permeability, fluorescence staining for CD31 and qRT-PCR for CD31 and VEGF was performed. SHH/*TP53*-mut tumors showed about 50% higher vascularization compared to Group 3 tumors (all vessels/FOV: 3.9 ± 0.2 vs. 2.6 ± 0.2; capillaries/FOV: 4.7 ± 0.3 vs. 3.1 ± 0.3; large vessels/FOV: 3.1 ± 0.2 vs. 2.0 ± 0.2; n = 10, *p* ≤ 0.01), (Figure 7a). After multimodal therapy with RT/DEC/ABC, the SHH/*TP53*-mut tumors were significantly less vascularized to 78.9 ± 7% (all vessels) and 79.1 ± 9% (capillaries) of control (n = 9; *p* ≤ 0.05). In Group 3 tumors a similar trend to reduced vascularization after multimodal treatment could be observed (all vessels/FOV: 2.6 ± 0.2 vs. 2.2 ± 0.1; capillaries/FOV: 3.1 ± 0.3 vs. 2.8 ± 0.2; large vessels/FOV: 2.0 ± 0.2 vs. 1.7 ± 0.2). Additionally, RT/DEC treatment decreased significantly the number of large vessels/FOV to 83.6 ± 7% of control in SHH/*TP53*-mut MBs (n = 5; *p* ≤ 0.05). However, CD31 and VEGF mRNA expression levels remained unchanged after therapy (Figure 7b).

### 2.9. Tumor Stemness Remained Unchanged after Multimodal Therapy

**Figure 8:**
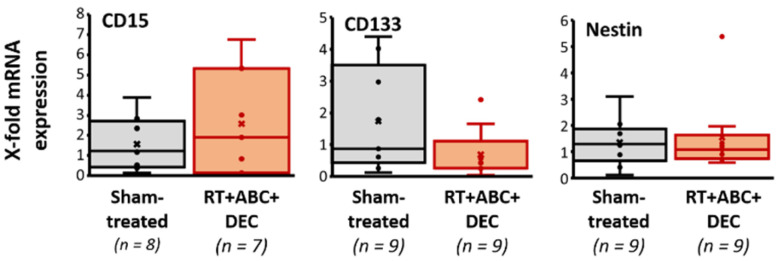
Stemness in Group 3 orthotopic medulloblastomas dependent on treatment of the MB-bearing mice with radiation therapy (RT), decitabine (DEC), and abacavir (ABC). The mRNA expression of potential human stem cell markers CD15, CD133, and nestin was determined by RT-PCR in Group 3 medulloblastoma tissue. Data are presented as box-and-whisker plots, numbers of analyzed animals/treatment group are indicated (n).

To evaluate therapy effects on the stemness of tumor cells, qRT-PCR for the potential human stem cell marker genes CD15, CD133, and nestin were conducted (Figure 8). No treatment-induced effects could be observed.

### 2.10. Invasion and Activation of Immune Cells

**Figure 9:**
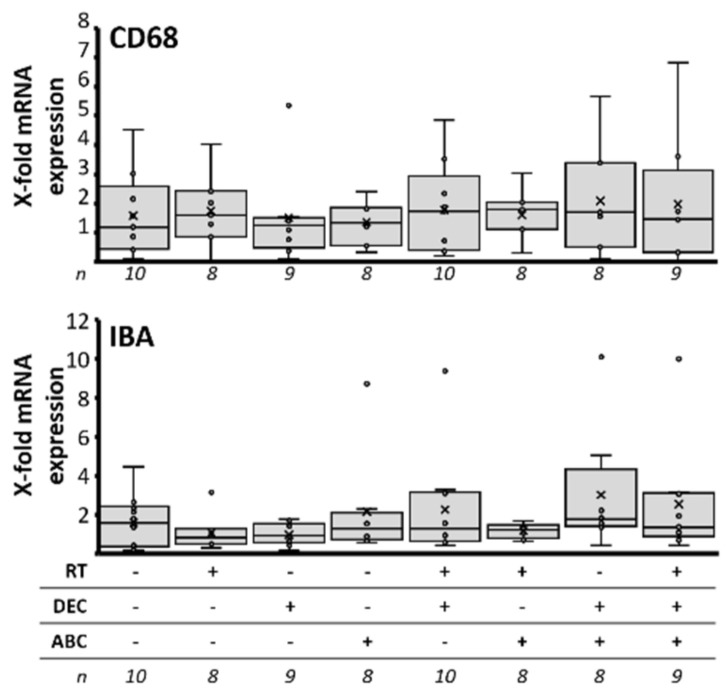
Immune response in Group 3 orthotopic medulloblastomas dependent on treatment of the MB-bearing mice with radiation therapy (RT), decitabine (DEC), and/or abacavir (ABC). The mRNA expression of murine CD68 (monocyte surface protein) and IBA (activated microglia) was determined by RT-PCR in Group 3 medulloblastoma tissue. Data are presented as box-and-whisker plots, numbers of analyzed animals/treatment group are given below (n).

To investigate if the therapy affects the amount of tumor-infiltrating macrophages or the activation of microglia, qRT-PCR of murine CD68 and IBA was performed (Figure 9). No treatment-induced effects could be observed.

### 2.11. Therapy-Induced Altered Gene Set Expression

**Figure 10:**
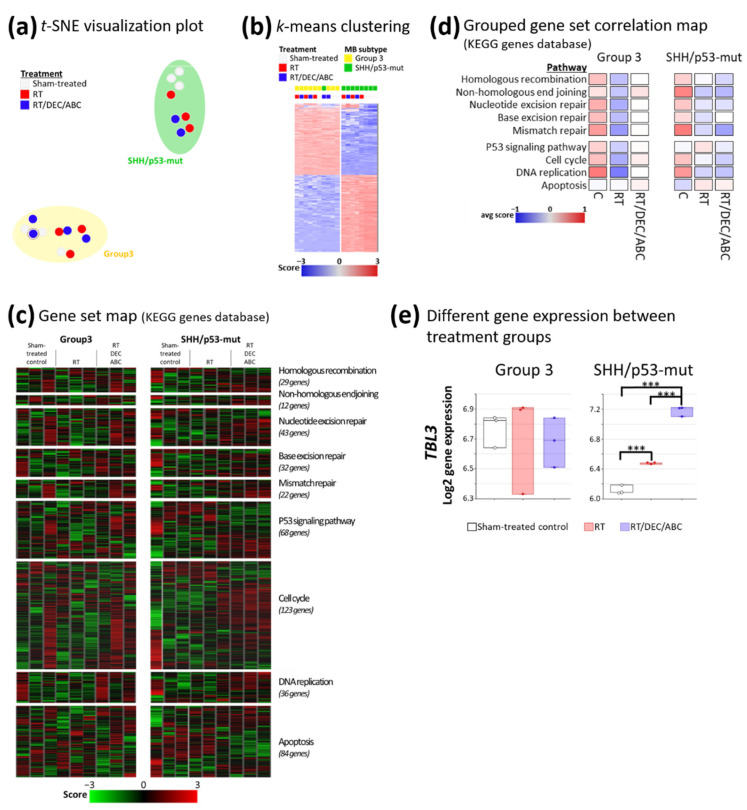
Gene expression array analyses in Group 3 and SHH/TP53-mut orthotopic medulloblastomas dependent on treatment of the MB-bearing mouse with placebo (sham-treated), RT, or RT/DEC/ABC. (**a**) Visualization of group clustering by t-distributed stochastic neighbour embedding (t-SNE) plot. The marked blue dot in the Group 3 MB cluster represents one animal belonging to SHH/*TP53*-mut MBs. (**b**) Meta-analysis by k-means (k = 2) clustering with log2/z-score transformation of expression data. Two clusters are formed by 1500 genes with the highest standard deviation (representing the highest expression changes compared to mean of all 11,648 genes on the array). (**c**) Detailed and (**d**) Grouped gene set map of treatment-relevant pathways using Kyoto encyclopedia of genes and genomes (KEGG). (**e**) Significantly different expressed TBL3 gene between treatment groups calculated by ANOVA with *p* ≤ 0.05 (sham-treated vs. RT or RT/DEC/ABC) and post-hoc Welch *t*-test; ***, *p* ≤ 0.001; three mice per treatment group.

Meta-analysis of gene expression (Figure 10a,b), showed a clear subtype-specific and even treatment-specific clustering (t-SNE visualization: Figure 10a and k-means, k = 2: Figure 10b). Gene set map analyses of treatment-related pathways using the Kyoto encyclopedia of genes and genomes (KEGG) revealed a diminished expression of gene sets (not significant) involving DNA repair, DNA replication, cell cycle, and p53 signaling by RT and RT/DEC/ABC; apoptosis gene set expression was not altered by treatment (Figure 10c,d). Changes in single gene expression between treatment groups (sham-treated vs. RT or vs. RT/DEC/ABC) were determined (Figure 10e). Thereby, only in SHH/*TP53*-mut MBs, the expression of TBL3 was significantly enhanced 1.2- and 1.8-fold in RT- and RT/DEC/ABC-treated mice.

## 3. Discussion

MB relapses are almost always fatal and occur in 30% of all cases [43]. Especially MB with *MYC* amplification and/or p53 dysfunction and/or Group 3 MBs show a higher degree of relapses with current treatment strategies [43,44,45,46]. However, as RT is a favorable prognostic factor enhancing relapse-free and overall survival in MB patients, innovative treatment strategies are urgently needed to enhance the RT-induced survival prolongation but avoiding additional systemic and local adverse effects.

Here, we investigated for the first time a multimodal therapy approach including RT, the epigenetic drug decitabine, and the telomerase inhibitor abacavir in a SHH/*TP53*-mutated and a *MYC*-amplificated Group 3 orthotopic PDX MB mouse model. Orthotopic PDX models maintain many features of the patient samples from which they derived [47,48,49,50] and are, therefore, currently the best preclinical model to analyze effects of innovative therapies. However, some limitations and differences compared to the human clinic have to be kept in mind [51]. Usually, MB patients undergo maximal tumor resection. In the mouse model, we start therapy with first BLI signal (tumor size 0.5–1 mm^3^), so the tumor burden might be different in mouse. Also, biological factors like radio- or chemosensitivity of normal tissue and number of tumor subclones can vary. In addition, we have to use an immunocompromised mouse model to investigate human tumor tissue, which implies a reduced or missing action of immune cells on tumor control propablity.

### 3.1. MRI Volumetry: T2-Weighted Maps versus Average Images

As pixel T2 relaxation times represented in a color-coded T2 map are directly comparable for different slices and measurements, advantages in T2 map compared to T2 average image analysis were presumed. Nonetheless, in a cohort of eight MB-bearing mice, no significant difference in tumor volumes determined from both contrast methods was identified (Figure 1). Due to time advantages in computation of T2 average images (three echo images per body slice) compared to T2 maps (pixel-wise extraction of T2 relaxation time from ten echo images), T2 average images were used for tumor volumetry of the following mice cohorts. Therefore, the 17-min MSME MRI sequence was maintained, although for future studies equivalently resolved T2 average images can be generated e.g., by a 4-min turbo/fast-spin echo sequence.

As can be seen from hyperintensity in T2 average images and quantified by means of the T2 map, T2 relaxation time of SHH/*TP53*-mut MB structures is clearly increased compared to healthy surrounding brain tissue (68 ms vs. 47 ms approx.). Slow T2 relaxation (long T2 relaxation time) occurs in free water-enriched environments as tumor tissues can be, due to its undifferentiated cell nature [52]. In such, the mobility of ^1^H-atoms is elevated and the local magnetic field affecting each ^1^H-atom fluctuates stochastically. As this is corresponding to a lack of magnetic field fluctuations, T2 relaxation progresses slowly in free water-enriched tissues. This effect is possibly the cause for hyperintensity in T2 contrast of the here examined SHH/*TP53*-mut MB since it manifested as the anaplastic histologically subtype with large, undifferentiated cells containing much cytoplasm [53].

### 3.2. BLI Total Flux Correlated Well with MRI-Based Tumor Volume

We confirmed the strong correlation of BLI measurements and MRI-based tumor volumetry (Figure 4) previously shown by others [54,55,56]. In contrast to Jost et al. [56] who found an excellent correlation only for small tumor volumes up to 20 mm^3^ in an orthotopic glioblastoma model, in our MB model volumes up to 40 mm^3^ correlate well. Above 40 mm^3^, we often observed a MRI signal within the ventricles that might be due to the infiltration of tumor cells or to the liquor stasis (hydrocephalus). BLI values showed a high inter-individual fluctuation, as also documented by Smith et al. [51], but did not reach a plateau until mouse euthanasia in contrast to data shown by Jost et al. [56]. Concerning time investment of both methods, mice handling and image generation takes about 20 min after optimization of both techniques. However, analyses of MR images is highly time-consuming as every single slice has to be processed separately (approx. 30 min per mouse per time point). Also, MRI is more expensive than BLI. In summary, to evaluate medulloblastoma tumor growth in mice up to a volume of 40 mm^3^, imaging by bioluminescence is an easy, quick, and reliable method.

### 3.3. Survival Benefit after Multimodal Therapy with RT, DEC, and ABC

Here, we could show for the first time in a preclinical model that RT combined with DEC and ABC significantly decreases tumor growth and enhances the survival of orthotopic SHH/*TP53*-mut and Group 3 MB-bearing mice (Figure 2). This is in line with our previous in vitro studies, demonstrating a significant RT-enhancing effect of DEC and ABC [29,33]. Thereby, the multimodal treatment reduced the clonogenic survival of MB cell lines 1500-fold and single treatments significantly reduced vital cell count and increased apoptosis and autophagy [33]. In addition we tested pre-, post- and pre/post-RT DEC administration in terms of radioadditive effects [29]. We found the pre/post regime most effective and adopted this in our in vivo experiments presented here.

The flattening of the BLI tumor growth curves by RT-including treatments in SHH/*TP53*-mut MB indicated growth stop or tumor cell death followed by regrowth one to four weeks (depending on treatment) after therapy stop (Figure 3d). Maximum growth delay was observed by RT/DEC/ABC, which is a strong evidence for the therapeutic action of this multimodal approach. However, the BLI growth measurements examined here represent a proof-of-concept experiment containing only one animal each group. The lack of an effect of RT alone on mouse survival (Figure 2) is probably due to the single dose treatment scheme applied here, as a significant survival benefit after fractionated RT of MB-bearing mice has been observed by others [51,57]. In our studies, Group 3 MB was slightly more sensitive to RT compared to SHH/*TP53*-mut MB resulting in a more prolonged median survival. Accordingly, others have shown the role of *TP53* loss of function mutations in radiation resistance of SHH-driven MB [57,58]. 

Low-dose DEC treatment, as applied here, acts mainly by epigenetic modulation than by induction of double-strand breaks (DSB) [29,33,59]. This could explain the missing benefit of the DEC monotherapy on mouse survival (Figure 2). However, in combination with RT, DEC significantly enhanced median mouse survival in both models, possibly by elevating the number of DSB paralleled by release of silenced tumor suppressor genes leading to an activation of RT-induced cell death pathways.

Also, ABC monotherapy showed no benefit on mouse survival (Figure 2) and tumor growth (Figure 3d) even though a significant effect on clonogenic cell survival was previously observed in MB cell lines [33]. Possibly telomerase inhibitor-induced shortening of telomeres might require longer treatment periods, about 50 cell divisions can be conducted before telomere shortening results in proliferation stop [60,61]. In combination with RT (Group 3) and RT/DEC (both models), a survival benefit of ABC could be demonstrated, possibly due to the induction of additional DSB by ABC [33] leading to DSB threshold crossing-causing cell death. In addition, the ABC-mediated downregulation of tumor cell proliferation [42] might avoid regrowth start after RT.

It is important to mention that the multimodal therapy applied here caused no or only weak adverse effects in the mice, evidenced by liver biopsies and mouse behaviour documentation. Accordingly, we previously reported that this multimodal treatment does not influence the number of neural progenitor cells in murine hippocampal slice cultures [33].

### 3.4. Less Proliferating Tumor Cells in the More Therapy-Resistant SHH/TP53-Mut MB Model after Multimodal Therapy with RT, DEC, and ABC

The overall proliferation index of sham-treated mice is similar in both models. However, the higher proliferative cell count ratio in high-to-low-proliferative areas in Group 3 MB might be partly responsible for the worse outcome compared to SHH/*TP53*-mut MB-bearing mice (Figure 2 and Figure 6a). So, it has been shown in MB patient samples that the proliferation index in so called hot spot areas (high density of Ki-67-positive tumor cells) is a significant prognostic predictor for poor survival [62]. In addition, other studies concerning the proliferation index in MB patient cohorts found a correlation of a high proliferation index with more aggressiveness and worse prognosis [63,64].

By analyzing the Ki-67 proliferation index in our tumor models after therapy, we found a significant reduction of proliferating cells only in the more therapy-resistant SHH/*TP53*-mut model after multimodal treatment. In line with this, an association between *TP53* gene alteration-mediated nuclear p53 accumulation and increased proliferation after RT has been observed in recurrent prostate carcinoma [65]. The lack of correlation to survival data suggests that the proliferating tumor cell fraction in our preclinical MB models does not or only in part influence the outcome. As the reduction was found on protein and not on mRNA level indicates that the therapy did not influence Ki-67 gene expression.

### 3.5. Reduced Vascularity after Multimodal Therapy with RT, DEC, and ABC in SHH/TP53-Mut MB

In sham-treated MB-bearing mice, we found a higher degree of vascularization in SHH/*TP53*-mut MB compared to the faster-growing and therefore more aggressive Group 3 MB (Figure 7a). Also in human samples, a subgroup-dependent vascular density was observed as high-risk SHH and Group 3 MBs showed a 4-times lesser amount of microvessels compared to the low-risk WNT MBs [66]. As both tumor models were at a similiar growth stage at euthanasia, possibly the Group 3 tumors are able to growth under nutrient/oxygen deficiency.

After the multimodal therapy with RT/DEC/ABC, we found a reduced vascularization in SHH/*TP53*-mut MB (and a trend in Group 3 MB). This goes along with the observed reduction of proliferating cells (Figure 6a) and suggests that a shortage of nutrients and/or oxygen might led to more quiescent, non-proliferative tumor cells [67]. However, this effect is not due to therapy-induced altered *VEGF* and *CD31* gene expression as mRNA levels remain stable.

### 3.6. No Enhancement of Stem Cell Marker mRNA Expression after Multimodal Therapy with RT, DEC, and ABC

Medulloblastoma cancer stem cells (CSC) are defined by the expression of several stem cell markers, including CD133 [68], CD15 [69], and nestin [70]. They often display radio- and chemotherapeutic resistance [71,72,73] and are therefore believed to be responsible for tumor recurrence [74]. The main causes of CSC resistance are an enhanced DNA repair capacity, ROS defense mechanisms, and a high self-renewing potential (reviewed in [75]).

RT is found to enhance the CSC fraction by (I) target the radiosensitive, proliferating non-CSCs, (II) reprogram normal tumor cells into CSC (iCSC) by increasing ROS and re-expression of stem cell regulators (reviewed in [76]) and (III) recruit quiescent CSC into a proliferative state after fractionated RT [77]. Interestingly, others reported CSC-targeting effects of DEC and ABC. Thereby, DEC reverses the chemotherapeutic resistance of colorectal and breast cancer CSCs by regulation of endogeneous microRNAs [78] and the telomerase-inhibiting capacity of ABC decreases the self-renewing ability of CSCs and induces their differentiation [42]. Importantly we could demonstrate here, that the multimodal therapy with RT, DEC, and ABC did not enhance the CSC fraction in orthotopic SHH/*TP53*-mut (by RT-PCR and gene expression array, data not shown) and Group 3 MB (by gene expression array, data not shown). Although an eradication of cancer stem cells and as a consequence thereof a reduction of relative stem cell amount marks the very best cancer therapy, the here observed unchanged CSC fraction suggests that also CSCs were efficiently targeted by this innovative treatment strategy (Figure 8).

### 3.7. No Therapy-Induced Expression of Immune Cell Markers

In general, human MBs show only a low expression of immune markers compared to other brain tumors [79]. It has been shown that MB exploit multiple immune evasion strategies including the downregulation of the recognition molecules MHC1 and CD1d [80]. Besides, subgroup-specific differences in quality and quantity of infiltrating immune cells are documented in immuno-competent mice and human patient samples [81,82]. Here, the investigated infiltration by macrophages/microglia and their activation of in Group 3 MB-bearing NSG mice lack a therapy-induced effect (Figure 9). This might be explained by the fact, that in this mouse strain, although macrophages are still present, they are functional defective [83] and miss the ability to detect the “eat me” signal on non-self cells [84].

### 3.8. Altered Expression of Genes Involved in Cell Cycle Regulation

Microarray arrays were performed on tumor tissue to examine treatment-induced changes in gene expression. Grouped gene set analyses suggested a downregulation of DNA repair and cell cycle pathways in MB tissue treated with RT or RT/DEC/ABC (Figure 10d). Significant expression alterations of the *TBL3* gene were found only in the SHH/*TP53*-mut model (Figure 10e).

Transducin beta-like protein 3 (TBL3) is found in zebrafish research to regulate cell cycle length during tissue differentiation [85]. Thereby, a loss of the *TBL3* gene causes slowing of the proliferation speed independent from p53 or cell death. In contrast, the here found significant therapy-induced enhancement of *TBL3* expression in MB tissue goes along with reduced tumor growth and survival benefit of treated SHH/*TP53*-mut MB-bearing mice. In accordance, in renal cancer a high protein level of TBL3 is also associated with a favorable prognosis [86].

The interpretation of missing mRNA expression changes after treatment is limited by the fact, that euthanasia/mRNA isolation took place weeks after finished treatment and that therefore possible transient changes could not be detected in this model.

## 4. Materials and Methods

### 4.1. Animals

NOD.Cg-Prkdc^scid^Il2rg^tm1Wjl^/SzJ mice, also known as NSG™ mice, originally from The Jackson Laboratory are highly immunocompromised mice with a high success rate for xenograft engraftment. Mouse breeding and housing was performed in the animal facility of the Medical Faculty, University of Leipzig according to European (Council Directive 2010/63/EU) and German (Tierschutzgesetz) guidelines for the welfare of experimental animals. NSG™ mice were housed in a 12/12 h light/dark cycle with access to high-energetic food and water *ad libitum*. All experiments had been approved in advance by the local authorities (Landesdirektion Sachsen TVV30/14, TVV31/18).

### 4.2. Patient-Derived Xenografts (PDX)

Patient-derived xenograft (PDX) cells of non-WNT/non-SHH, Group 3 and of SHH/*TP53*-mutated medulloblastoma were generated at the German Cancer Research Center (DKFZ) Heidelberg, Germany. The Group 3 MB cells harbor a *MYC* amplification. The SHH/*TP53*-mut MB cells harbor a *MYCN/GLI2* amplification and were additionally GFP/luciferase-transfected (pGreenFire-CMV). Both subtypes were histologically anaplastic.

### 4.3. Orthotopic PDX Mouse Model

For orthotopic tumor cell transplantation, about 8 × 10^4^ PDX cells were injected intracranially using a motorized stereotactic frame (Stoelting™). Therefore, frozen cell solution was thawed and carefully resuspended in NeuroCult™ NS-A Basal Medium (human) + Proliferation Supplement (Stemcell Technologies, Vancouver, Canada) and kept on ice until injection.

NSG™ mice were narcotized (0.5 mg/kg medetomedine, 5 mg/kg midazolam, 0.05 mg/kg fentanyl, i.p.) and fixed in the stereotactic frame using blunt ear bars. Body temperature was kept between 36–38 °C using a rectal probe connected with a heating pad. The back of the head was shaved, disinfected and locally anesthetized (lidocaine hydrochloride solution). Using a scalpel, a 1-cm-long incision was made and the scalp was carefully pushed aside. At −1.5 mm lateral and 2 mm caudal of lambda, a 0.7 mm hole was drilled using a Stoelting™ micro drill. Injection of 3 µL tumor cell solution was conducted using a Hamilton precision syringe (model 702 RN) with a 26G needle (length: 33 mm; point style: 4, 30°). Needle was inserted at 2.5 mm depth below the dura mater and cell solution was injected at 0.5 µL/min. After finished injection, needle was lifted 0.5 mm and left in place for another 3 min to allow for pooling of solution and to avoid capillary effect. Scalp was closed using tissue glue (Histoacryl^®^, B.Braun, Melsungen, Germany) and disinfected. Analgesia (5 mg/kg carprofen s.c.) and anesthesia antagonists (2.5 mg/kg atipamezole, 0.5 mg/kg flumazenil, 1.2 mg/kg naloxone, i.p.) were applied.

Mice were given analgesia (daily 5 mg/kg carprofen, i.p.) for the following 5 days and kept in individual housing for 7 days to allow for wound healing. After, mice were group-housed, with daily health monitoring and weighting at least twice a week until showing signs of humane endpoints (written in the animal experimental proposal authorized by the Landesdirektion Sachsen TVV30/14; TVV31/18) immediately leading to euthanasia by CO_2_ anesthesia and decapitation.

Tumor-bearing brains were removed, immersed in cold phosphate-buffered saline (PBS), scissored as a whole, and tumor cells were harvested using a Pasteur pipette for homogenization of tumor tissue. Tumor cells were separated using a 50 µm cell strainer (CellTrics^®^, Sysmex, Kobe, Japan), resuspended and counted in PBS. Tumor cells were then aliquoted in NeuroCult™ NS-A Basal Medium (human) + Proliferation Supplement (Stemcell Technologies) + 10% DMSO and frozen in liquid nitrogen until transplantation. One cell batch (batch = cells harvested out of one mouse) was used for tumor inoculation of all following mice within all studies concerning one MB subtype mouse model.

### 4.4. Treatment of MB-Bearing Mice with Decitabine (DEC) and Abacavir (ABC)

Drug solutions (2 µL/g mouse body weight; 50 mg/kg/d abacavir and 0.1 mg/kg/d decitabine) were applied intraperitoneally on both sides using 30G insulin syringes (Omnican^®^50, B. Braun) daily for 14 consecutive days (d1–14). Solutions were prepared freshly every two days using 0.9% NaCl (B. Braun) for abacavir sulfate (MilloporeSigma, Burlington, MA, USA; work solution 25 mg/mL) and sterile water (B.Braun) for decitabine (Abcam, Cambridge, UK; work solution 0.05 mg/mL) and stored at 4 °C. Placebo solutions without drugs were applied in the relevant mice.

### 4.5. Radiation Therapy of MB-Bearing Mice

Local single-dose whole brain irradiation (WBI) was applied at day 8 of drug injection. Therefore, a 200 kV orthovoltage X-ray generator (Gulmay D3225, Gulmay GmbH, Krefeld, Germany) with a dose rate of 1 Gy/min was used. Mice were irradiated using a custom-made radiation cage that allows an anesthesia-free local head irradiation.

### 4.6. Magnetic Resonance Imaging (MRI)

To determine the optimal therapy start for Group 3 animals, MRI was conducted at Helmholtz Centre Dresden-Rossendorf, Research site Leipzig, on a small animal 1 Tesla magnet (PET/MR 1Tesla; nanoScan^®^; MEDISO Medical Imaging Systems, Budapest, Hungary) using a volumic head coil. Animals were anaesthetized using isoflurane (1.8%, 0.35 L/min) delivered in a 60% oxygen/40% air mixture and the body temperature kept at 37 °C with a thermal bed system during the entire acquisition time. The software Nucline 2.01 (MEDISO Medical Imaging Systems, Budapest, Hungary) was used for acquisition and analyses of the data. T2-weighted sequence (Fast Spin Echo; TR/TE: 4377/88.5 ms; FA: 180°; NEX: 2; Matrix: 256x256; ST: 0.8 mm) was used for tumor monitoring.

For SHH/*TP53*-mut MB animals, MRI was conducted at the IZI Leipzig in a 7 Tesla small animal MRI scanner (Pharmascan 7 T, Bruker, Billerica, MA, US) using a transmit-receive volume head coil (T20061V3, Bruker). After BLI, anaesthesia was nearly maintained by transferring the animal directly into the MRI animal bed, equipped with a nose cone anaesthesia delivery system. 1–2% isoflurane in oxygen (flow rate 0.5 L/min) were supplied and depth of anaesthesia was continuously monitored with the help of a respiration pressure pad under the animal abdomen signaling to the Bio-SAM respiration monitoring software (SA Instruments, Stony Brook, NY, USA). Body temperature stability was ensured by a tempered water system inside the animal bed (T12555, Bruker). To adjust animal positioning within the scanner, one slice for each orthogonal plane was acquired in a FLASH-Localizer-sequence. Using a multi-slice-multi-echo (MSME) sequence with a repetition time (TR) of 4000 ms, each coronal slice was imaged in two proton-density (PD)-weighted and eight T2-weighted images (echo times (TE): 12.5, 29.2, 45.8, 62.5, 79.2, 95.8, 112.5, 129.2, 145.8 and 162.5 ms) which corresponds to an echo spacing of 16.67 ms. The entire mouse brain was covered by 20 slices of 0.8 mm width without a gap and a field of view of 20 × 20 mm mapped in a 256 × 256 pixel matrix. By conducting MSME measurements with a total scan time of approximately 17 min, we were able to access pixelwise T2 relaxation times which were then plotted in T2 relaxation time maps (T2 map) for each slice.

### 4.7. MRI Image Processing and Tumor Volumetry Based on T2-Weighted Average Image

To evaluate suitability for volumetric analysis of MB, T2 average image as well as T2 map were calculated for each tumor-affected coronal slice.

Computing the T2 average image, three one-slice echo images captured at echo times of 45.8, 62.5 and 79.2 ms were averaged pixel by pixel in Matlab (version R2015b, MathWorks, Natick, MA, USA) to reduce image noise. Additionally, the span of average intensities represented by the given gray scale could be manually adapted to enhance tumor contrast slice-individually.

To calculate a slice-corresponding T2 map, the image intensities of all 10 echo images of the tumor-affected slice were collected into a 256 × 256 × 10 matrix by calling the function *dicomread* (Image Processing Toolbox) in Matlab. In a for loop through each pixel of the 256 × 256 image matrix, intensities of all 10 echo images were extracted and their time course was fitted by the exponential function describing a T2 relaxation process (Equation (1)).

(1) $$S(\mathrm{TE})=S_{0}\cdots e^{-\frac{TE}{T2}}$$

To identify the best fit function, the sum-squared error cost function was minimized with the Matlab function *fminsearch* using a maximum number of 10 million iterations. By extracting the T2 relaxation time (parameter T2 in Equation (1)) from the appropriate fit function of each pixel, a matrix of T2 relaxation times was generated. Those were then mapped to the colormap *jet* color range with T2 > 120 ms allocated to the maximum color value, yielding a high tumor-contrasted T2 map. Processing time for one T2 map was approximately 9.5 min using a standard costumer PC (Windows 10, 64 bit, Intel Core i5 3rd Gen. @ 2.5 GHz, 8 GB RAM).

**Figure 11:**
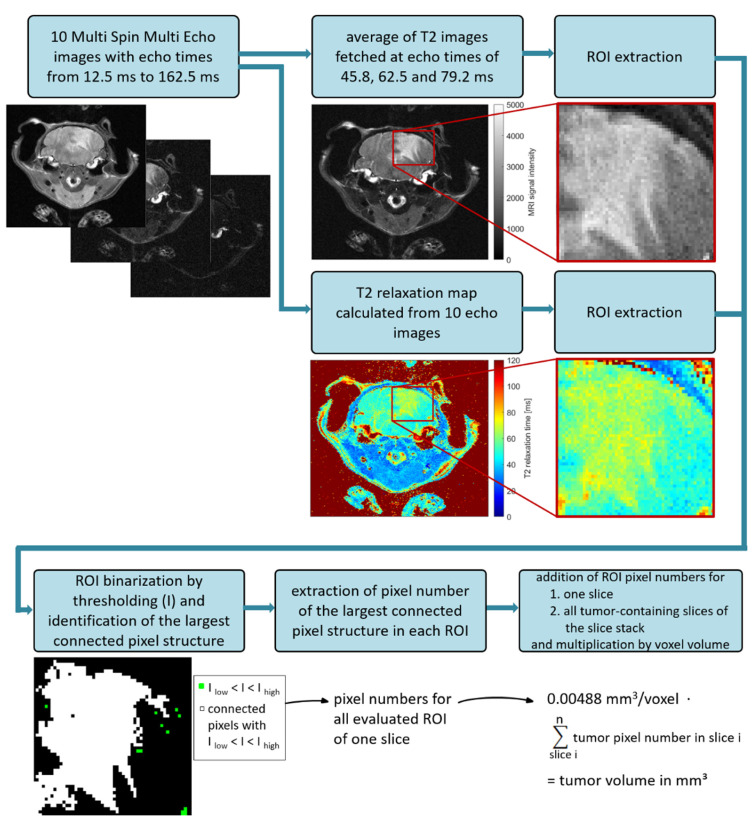
Image processing chain to extract tumor volume in an example slice with one ROI.

Volumetric analysis of tumor-affected slices in both contrast methods, T2 average image and T2 map, were carried out semiautomatically in Matlab using an own-built thresholding method illustrated in Figure 11. Depending on the cross-sectional tumor composition, one to five region(s) of interest (ROI) were defined by the help of data tips in the Matlab figure tools. Subsequently, ROI were binarized applying ROI-individual upper and lower thresholds for gray scale intensities (T2 average image) or plotted relaxation times (T2 map), respectively. ROI size and thresholds were adapted until proper segmentations of tumor regions were achieved. The largest connected pixel structure (connectivity of 8) was identified for each binarized ROI using the function *bwconncomp* (Image Processing Toolbox). Pixel numbers of the largest connected structure in each ROI were first added slice-wisely and then for all tumor-affected slices. By multiplying the total tumor-representing pixel number by the volume per voxel (0.0048 mm^3^/voxel), the final tumor volume for one MRI measurement was obtained.

To compare volumetric MRI analysis for the contrast methods T2 map and T2 average image, the paired differences of tumor volume were plotted against the corresponding paired volume means in a Bland-Altman plot. Such allows for an assessment of systematic deviations of the scattered differences as a function of the tumor load (mean tumor volume). Furthermore, a paired sample *t*-test was carried out to examine the hypothesis of mean differences between both observation sets are zero. Normal distribution could be assumed as the central limit theorem applied (n > 30).

### 4.8. Bioluminescence Imaging (BLI)

Beginning at week 1 post tumor cell injection, BLI of SHH/*TP53*-mut MB-bearing mice was performed weekly using the Xenogen IVIS^®^ Spectrum Imaging System and Living Image 3.2 software at the IZI or at the animal facility of the Faculty of Medicine (MEZ), University of Leipzig, Germany. 

Therefore, mice were anesthetized by inhalable narcosis (IZI: Xenogen XGI-8 Gas Anesthesia System; induction with 2.5–3% isoflurane in oxygen; flow rate 1.2 L/min; maintenance 1.5–2% isofluorane in oxygen) or antagonizable narcosis (MEZ: induction 0.5 mg/kg medetomedine, 5 mg/kg midazolam, 0.05 mg/kg fentanyl, i.p.; recovery 2.5 mg/kg atipamezole, 0.5 mg/kg flumazenil, 1.2 mg/kg naloxone, i.p.). BLI was performed always 9 min after i.p. injection of 150 mg/kg D-luciferin (BioVision). BLI was conducted with medium binning of 8, a fully opened entrance pupil (f/1), and a field of view of 12.6 × 12.6 cm. Tumor size proportional amount of photons was first measured in a 60 s measurement, followed by a 30 s measurement (at min 10). Subsequently, an auto exposure measurement was initialized (at min 11), where measurement time was automatically adjusted between 5 and 120 s depending on the amount of incoming photons during a pre-measurement. Photons per second (p/s, total flux) were used as analyzed parameter. Total flux of a caudal background region (BKG) was determined for each mouse and subtracted from total flux of tumor region of interest (ROI). For BLI data analysis, the highest value out of the three measurements of each time point was normalized to total flux of week 1 post tumor cell injection.

### 4.9. Correlation Analysis of MRI and BLI Values

To correlate total flux measured by BLI and tumor volume determined by MRI, the logarithmic regression model was used (IBM^®^ SPSS^®^ Statistics 25). Therefore, the raw values of total flux and tumor volume were transformed using the decadic logarithm, log_10_(*x*), and plotted in a linear/linear diagram. Then, a linear regression curve, the coefficient of determination (R^2^), the Spearman’s rank correlation coefficient (ρ), and the *p* value for significance of correlation were calculated. 

Expected values of total flux were calculated by inverse transformation of the linear regression formula into the logarithmic regression (Equation (2)).

(2) $$\begin{array}{c} y=a+b\cdot x\\ \log_{10}y=\log_{10}a+b\cdot \log_{10}x\\ \log_{10}y=\log_{10}a+\log_{10}x^{b\;}\;\;\;\;\;\left|10^{\log_{10}(y,\;a,\;x^{b})}\right|\\ y=a\cdot x^{b} \end{array}$$

Absolute residuals of total flux were calculated by subtracting the expected from the observed values (Equation (3)). Relative residuals were calculated by dividing absolute residuals by observed values (Equation (4)).

(3) $$abs.\;residual=value\;\left(observed\right)-value\;\left(expected\right)$$

(4) $$rel.\;residual=\frac{abs.residual}{value\;\left(observed\right)}$$

### 4.10. Start of the Multimodal Therapy

To determine the therapy start, weekly measurements by non-invasive imaging (MRI, BLI) on at least 8 mice were conducted for each MB subtype. The time point when the tumor is visible for the first time was set as therapy start (day 21 for Group 3, day 7 for SHH/*TP53*-mut).

### 4.11. Survival Analysis

Mice have to be euthanized due to human endpoints (weight loss > 20%, severe bulky head, serious neurologic deficits, pain) specified in the animal experiment proposal authorized by the Landesdirektion Sachsen (TVV30/14 and TVV31/18).The time from day of tumor cell injection to euthanasia (survival time) was analyzed using Kaplan-Meier method (IBM^®^ SPSS^®^ Statistics 25). The survival curves were statistically compared by the log rank test (IBM^®^ SPSS^®^ Statistics 25). Median survival is defined as time point when half of mice are still alive. Significant differences in median survival compared to sham- or radiation-treated group were calculated by two-sided Mann-Whitney test (IBM^®^ SPSS^®^ Statistics 25).

### 4.12. Preparation and Hematoxylin/Eosin Staining of Mouse Brain and Liver

Immediately after euthanasia, brain was extracted from skull cavity, briefly rinsed in cold PBS, embedded in Tissue-Tek^®^ O.C.T.™ Compound using Cryomold^®^ embedding bowls, frozen in liquid nitrogen-cooled isopentane, and stored at −80 °C until further use. For histological analyses, 10-µm-thick cryosections were prepared every 100 µm in the cerebellum area containing tumor and hematoxylin/eosin staining performed. For RT-PCR analyses, intermediate sections were preserved.

Livers were extracted from abdominal cavity, fixed in 4% formaldehyde for at least 24 h, embedded in paraffin at the Department of Neuropathology Leipzig, cut into 4 to 10-µm-thick sections, and stained with hematoxylin/eosin. Pathological diagnosing was conducted by Prof. Müller, Department of Neuropathology, University of Leipzig, Germany.

### 4.13. Immunostaining of Ki-67 and CD31

Cryosections were fixed with ice-cold 70% ethanol for 10 min and with absolute ethanol for further 10 min, followed by a permeabilization step with 0.5% Triton-X 100 (Sigma-Aldrich) in PBS for 5 min. For Ki-67 staining, slices were rinsed with PBS and stained with Mouse to Mouse HRP (AEC) Staining System (Dianova) according to manufacturers’ instructions. Primary anti-Ki-67 antibody (Dianova; rabbit anti-human Ki-67, Clone RM3600, 1 mg/mL, 1:200) was diluted in 2% normal goat serum in PBS. After counterstaining with Mayer’s hemalum solution, slices were mounted with glycerol gelatin (Dr. K. Hollborn & Söhne GmbH). For each mouse, three sections were stained and counted with 6 fields-of-view (FOVs) per mouse at 400-fold magnification. Thereby, three FOVs showing a high density (higher than average; ‘high-proliferative areas’) and three FOVs showing a low density (lower than average; ‘low-proliferative areas’) of Ki-67-stained cells were analyzed manually at a Zeiss Axiolab microscope. 

For CD31 staining, slices were immersed with 10% normal goat serum (Invitrogen) + 0.25% Triton-X 100 in PBS for 30 min, rinsed with PBS, and incubated with anti-CD31 primary antibody (eBioscience; rat IgG2a anti-mouse CD31, 0.5 mg/mL, 1:100) diluted in 2% normal goat serum + 0.25% Triton-X 100 in PBS at 4 °C overnight. After three washes with PBS for 5 min, slices were incubated with Alexa Fluor^®^ 488-conjugated secondary antibody (Invitrogen; goat anti-mouse IgG antibody F(ab)2, 2 mg/mL, 1:800) diluted in 2% normal goat serum + 0.25% Triton-X 100 in PBS for 1 h, washed three times with PBS for 5 min, stained with DAPI (5 µg/mL in aqua dest.) for 5 min, and mounted with Mowiol^®^ 4-88/DABCO (Roth). To quantify CD31-positive capillaries and larger vessels, 12 fields of view (FOVs) per mouse at 400-fold magnification were counted manually.

Data of each treatment group were statistically analyzed compared to sham- or irradiation-treated group by two-sided Mann-Whitney test (IBM^®^ SPSS^®^ Statistics 25).

### 4.14. Gene Expression RT-PCR Analyses

Total mRNA was extracted from tumor-bearing brain tissue by homogenization using RNAzol^®^RT (Sigma-Aldrich; at least 0.2 mL/10^6^ cells) following manufacturers’ protocol. MRNA concentration was measured spectrophotometrically (NanoVue™Plus Spectrophotometer, GE Healthcare, Chicago, IL, USA) and cDNA synthesized using random hexamer primers (Thermo Scientific™, Thermo Fisher Scientific, Waltham, MA, USA) and Omniscript RT Kit (Quiagen, Hilden, Germany). Quantitative real-time PCR (qRT-PCR) was performed on a Rotor-Gene 3000 cycler (Corbett Life Science, Quiagen, Hilden, Germany) using the Takyon™ No Rox Probe MasterMix (Eurogentec, Serain, Belgium) to amplify murine CD31, VEGF, IBA, CD68, and human CD15, CD133, nestin, Ki-67, and the house-keeping genes human/murine ß2-microglobulin and HPRT1. Primer/probe sets are listed in Table 2. Design of exon-spanning primer pairs and probes was executed using the PrimerQuest™ Tool (Integrated DNA Technologies, Coralville, IA, USA). To identify possible crossreactivities to human or mouse nucleotide sequences, all primer/probes were checked using the Blast online tools at the National Centre for Biotechnology Information (NCBI) (https://blast.ncbi.nlm.nih.gov/; assessed on 13 December 2017). Expression levels were normalized against both house-keeping genes using the maximal efficiency sensitive model [87] and to the normalized expression level of the sham-treated corresponding mouse within one treatment group (= relative gene expression). Data of each treatment group were statistically analyzed compared to sham- or irradiation-treated group by two-sided Mann-Whitney test (IBM^®^ SPSS^®^ Statistics 25). Statistical comparisons between the sham-treated control groups of both MB subtypes were done by Student’s *t*-test.

### 4.15. Gene Expression Microarray Analyses

Gene expression data of tissue samples from sham-, RT-, and RT/DEC/ABC-treated mice (n = 3 each group) were generated at the German Cancer Research Center Heidelberg, Germany using the Affymetrix U133 Plus 2.0 array (Affymetrix Inc., Santa Clara, CA, USA). Data were normalized by the MAS5.0 algorithm (GCOS software, Affymetrix Inc.), analyzed and visualized using the R2: Genomics Analysis and Visualization Platform (http://r2.amc.nl; assessed on 23 August 2021). Thereby meta analyses: *t*-SNE, *k*-means clustering (Log2 z-score transformation, 2 clusters of the 1500 highest 2log standard deviation entries), gene set mapping (gene set collection: Kyoto encyclopedia of genes and genomes, KEGG; z-score transformation) and differential expression between multiple group analyses (ANOVA, Log2 transformation, *p*-value cutoff: 0.05) were performed.

### 4.16. Statistics

The used statistical tests are specified in the respective method part. Differences were considered statistically significant at *p* ≤ 0.05 (*; #) or very significant at *p* ≤ 0.01 (**; ##).

## 5. Conclusions

Here, we showed for the first time in a preclinical model that the multimodal therapy combining RT, DEC, and ABC improves the survival of ortothopic SHH/*TP53*-mut and Group 3 MB-bearing mice. Thereby, the tumor growth was inhibited, but regrowth started after treatment stop as observed by BLI and MRI. Therefore, longer treatment periods including fractionated RT might be required to improve the survival further. A correlation between tumor proliferation index or vascularization and mouse survival could not be verified, suggesting that additional molecular mechanisms are responsible for the observed survival benefit. Stem cell marker expression analysis indicated that the treatment targets tumor cells and cancer stem-like cells equally. Gene expression array analyses suggested a therapy-induced regulation of genes involved in DNA repair and cell cycle regulation. No therapy-induced adverse effects were observed in the mice, which is an important prerequisite for the clinical application of this multimodal therapy approach.

## Figures and Tables

**Table 1:** Survival analyses: Number of tumor-bearing animals and therapy groups.

Therapy	Group 3 (n = 77)	SHH/*TP53*-Mutated (n = 80)
Sham-treated	10	10
RT	9	10
DEC	10	10
ABC	10	10
RT/DEC	10	10
RT/ABC	9	10
DEC/ABC	10	10
RT/DEC/ABC	9	10

**Table 2:** Primer/probes sets for qRT-PCR.

Species*/Gene *M…Mouse; H…Human *NCBI Reference Sequence*	Forward Primer (5′-3′)	Probe (5′-3′)	Reverse Primer (5′-3′)
mVEGF *NM_009505.4*	AACGATGAAGCCCTGGAGTG	YakimaYellow™-CGTGCCCACGTCAGAGAGCAACATCA-BHQ1	ATGTGCTGGCTTTGGTGAGG
mCD31 *NM_001032378.2*	CCAGGTGTGCGAAATGCTCT	YakimaYellow™-AAGGACATGCCATAGGCATCAGCT-BHQ1	GGTGGTAAGTGATGGGTGCAG
mIBA *NM_019467.2*	GCAGGGATTTGCAGGGAGGA	HEX-CCAGCCTCTCTTCCTGCTGGGCC-BHQ1	TGGACGGCAGATCCTCATCA
mCD68 *NM_001291058.1*	GCTGTGGAAATGCAAGCATAG	HEX-AGGCTACAGGCTGCTCAGCTGCC-BHQ1	GAGAAACATGGCCCGAAGT
hNestin *NM_006617.2*	AGGAGAAACAGGGCCTACA	YakimaYellow™-CACCTCAAGATGTCCCTCAGCCTG-BHQ1	AGGAGGGTCCTGTACGTG
hCD133 *NM_001145852.2*	ATGAAACTCCAGAGCAAATC	YakimaYellow™-TACAACACTACCAAGGACAAGGCG-BHQ1	GTCTCAGTCGGTCAAGAA
hCD15 *NM_002033.4*	GGGTTTGGATGAACTTCG	YakimaYellow™-AGAGCGTCCAGTTGAAGAGGTTAC-BHQ1	GGGTAGAGGTAGCCATAAG
hKi-67 *NM_002417.5*	CAGAATGGAAGGAAGTCAAC	YakimaYellow™-AATACGTGAACAGGAGCCAGCA-BHQ1	TTCTCATCAGGGTCAGAAG
mß2-microglobulin *NM_009735.3*	TGAGACTGATACATACGCCTGCA	HEX-ATGGCCGAGCCCAAGACCGTC-BHQ1	GATGCTTGATCACATGTCTCGATC
hß2-microglobulin [88]	TGACTTTGTCACAGCCCAAGATA	BHQ1-TGATGCTGCTTACATGTCTCGATCCCA-HEX	AATCCAAATGCGGCATCTTC
hHPRT1 *NM_004048.4*	GACTTTGCTTTCCTTGGTCAG	YakimaYellow™-CCAAAGATGGTCAAGGTCGCAAGC-BHQ1	TGGCTTATATCCAACACTTCGT
mhprt1 *NM_009735.3*	AAACTTTGCTTTCCCTGGTTAA	YakimaYellow™-ACCAGCAAGCTTGCAACCTTAACC-BHQ1	CCTGTATCCAACACTTCGAGA

## Data Availability

The datasets used and/or analyzed during the current study are available from the corresponding author on reasonable request.
